# Case report: Polymorphous low-grade neuroepithelial tumor of the young and supratentorial ependymoma diagnosed in an adult male

**DOI:** 10.3389/fneur.2024.1482832

**Published:** 2024-11-08

**Authors:** Cynthia Y. Xu, Craig A. Beers, Jian-Qiang Lu, Crystal L. Hann, Ronald C. Ramos

**Affiliations:** ^1^Division of Neurology, McMaster University, Hamilton, ON, Canada; ^2^Faculty of Health Sciences, McMaster University, Hamilton, ON, Canada; ^3^Division of Radiation Oncology, Juravinski Cancer Centre, Hamilton, ON, Canada; ^4^Department of Pathology and Molecular Medicine-Neuropathology, McMaster University, Hamilton, ON, Canada

**Keywords:** PLNTY, ependymoma, neuro oncology, radiation oncology, adult neurology

## Abstract

Polymorphous low-grade neuroepithelial tumor of the young (PLNTY) is a rare central nervous system (CNS) pathology predominantly observed in the pediatric population. Ependymomas also exhibit a peak incidence in early childhood, with rare presentations after early adulthood. In this report, we describe a rare case of a 41-year-old man diagnosed sequentially with a polymorphous low-grade neuroepithelial tumor of the young, followed by a supratentorial ependymoma within a year. He underwent tumor resection for both tumors, as well as adjuvant radiation therapy for the ependymoma. Despite these interventions, he ultimately succumbed to tumor progression and postoperative complications. Currently, no genetic syndromes are known to link these two primary CNS tumors. Two commonalities at the chromosomal and cellular level include histone gene H3F3A mutations and positive glial fibrillary acidic protein staining on immunohistochemistry. To the best of our knowledge, this unique dual pathology has not been previously described in the literature, making this case an avenue for further investigation and research into connections between these two distinct CNS pathologies.

## Introduction

Polymorphous low-grade neuroepithelial tumor of the young (PLNTY) is a relatively new classification of low-grade neuroepithelial tumors first described in 2017 (1).

PLNTY is a rare central nervous system (CNS) pathology that is primarily observed in children and young adults, typically presenting with headaches, focal neurological deficits, and, most commonly, seizures (2, 3). Histopathologically, PLNTYs are characterized by infiltrative growth patterns and oligodendroglioma-like components, often with astrocytic and ependymocytic components (1). On immunohistochemistry, PLNTYs demonstrate immunopositivity on CD34 testing and typically will demonstrate genetic abnormalities involving either B-Raf proto-oncogene (BRAF) or fibroblast growth factor receptors 2 and 3 (FGFR2, FGFR3) (1). As previously discussed, PLNTY typically presents in children and young adults. However, there are select case reports of adult presentations: one case in a 50-year-old woman (4) and a second in a 37-year-old man (5).

The primary management of PLNTY is gross total resection (GTR), which typically follows an indolent course with symptoms resolving postoperatively (3).

Similar to PLNTY, intracranial ependymomas exhibit a peak incidence in early childhood, with rare presentations after the age of 40 (6, 7). Clinical features vary depending on the location of the tumor and may include increased intracranial pressure, focal neurological deficits, and seizures. In pediatric cases, approximately 90% of ependymomas present intracranially (7, 8), whereas in adults, approximately 65% of ependymomas are found in the spinal cord (9). The WHO classification of CNS tumors categorizes and grades ependymomas primarily by their anatomical location and molecular subgroup (10). Anatomically, ependymoma is classified into spinal, infratentorial, and supratentorial subgroups, while molecularly, they are classified as follows: supratentorial ependymoma with ZFTA fusion (ST-EPN-ZFTA), supratentorial ependymoma with YAP1 fusion (ST-EPN-YAP), supratentorial ependymoma without ZFTA or YAP1 fusion, posterior fossa ependymoma group A (PF-EPN-A), and posterior fossa ependymoma group B (PF-EPN-B) (11). Management of intracranial (supra and infratentorial) ependymomas is typically maximally safe total resection followed by adjuvant radiotherapy and chemotherapy as indicated, following a multidisciplinary approach (12, 13).

We present herein the case report of a 41-year-old man who presented and was managed for a right frontal PLNTY, then subsequently was found to have a left frontal supratentorial ependymoma.

## Case report

**Figure 1:**
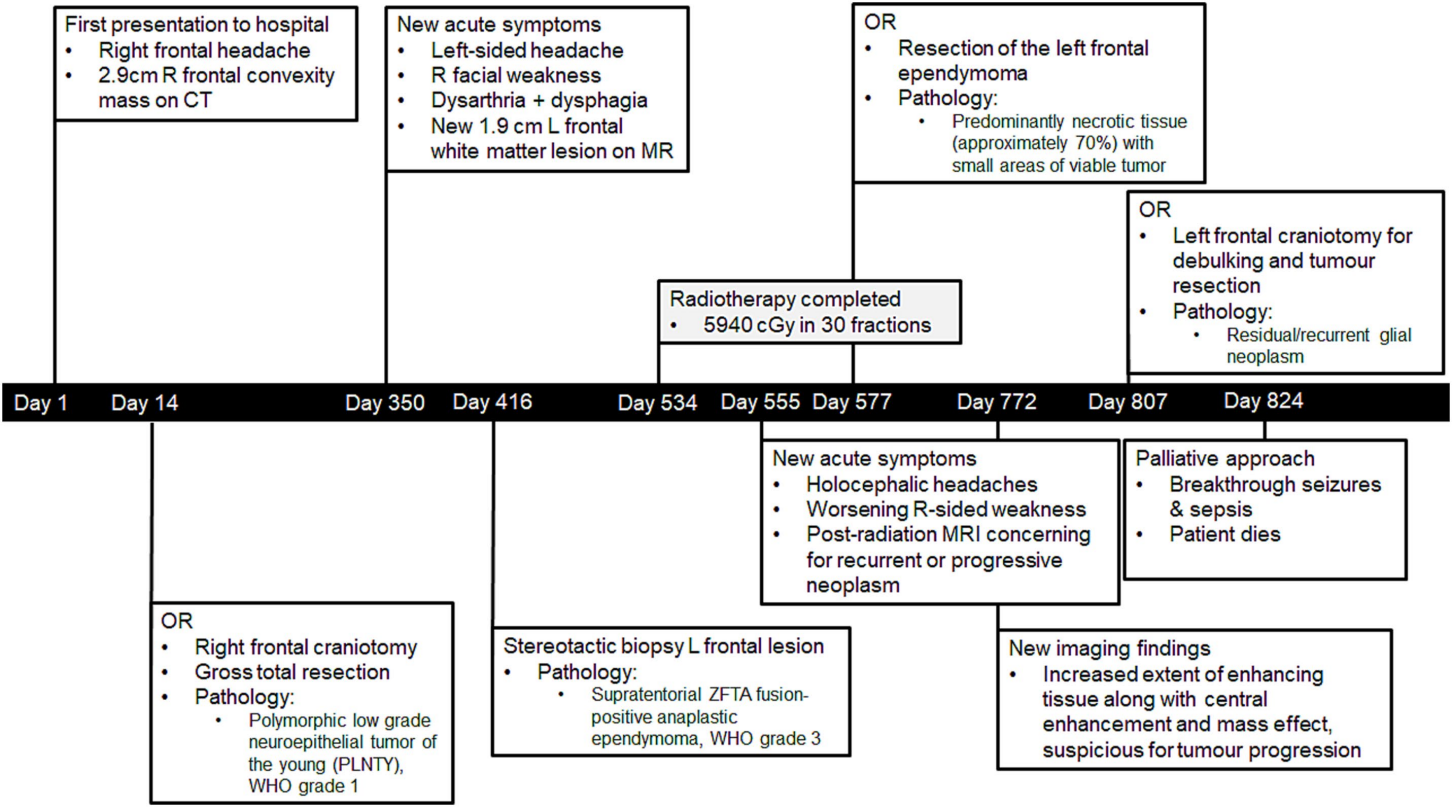
Timeline of Mr. A’s diagnoses and care.

A 41-year-old man (Mr. A) presented to the hospital in January 2021 following a first-time tonic–clonic seizure. He also reported a new right frontal headache that had started 1.5 weeks prior to admission (Figure 1). He had no significant past medical history. From a social perspective, he worked at a car dealership operating heavy machinery, denied using tobacco or recreational substances, and lived in a moderately sized city (population of approximately 140,000). Mr. A was married, and he and his wife had three children.

**Figure 2:**
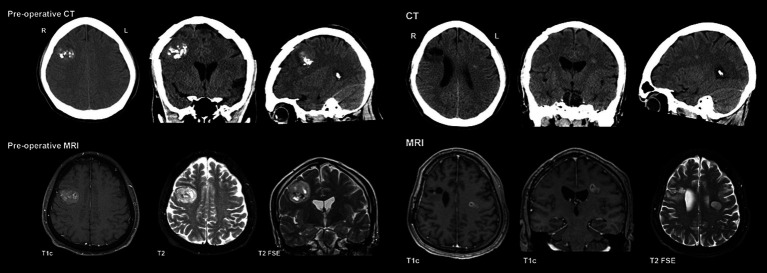
Preoperative CT/MRI for PLNTY & Preoperative CT/MRI for ependymoma.

Initial imaging (Figure 2) included a CT head, which demonstrated a lesion in the high right frontal convexity measuring 2.8 × 3.2 cm × 2.9 cm, characterized by popcorn-like calcifications, absence of fat tissue elements, and mild adjacent parenchymal edema. Subsequent MRI of the brain (Figure 2) localized the mass to the right middle frontal gyrus, demonstrating a heterogeneous high T2 signal with intralesional cystic/necrotic components and areas of T1 & T2 hyperintensity in keeping with intralesional calcification. After multidisciplinary assessment and discussion, surgical resection was recommended.

Mr. A underwent a right frontal craniotomy for tumor resection 14 days after initial presentation and imaging. The procedure was deemed to be a gross total resection. Pathological examination confirmed the tumor to be a polymorphic low-grade neuroepithelial tumor of the young (PLNTY), WHO grade 1. Immunohistochemistry showed CD34 and GFAP immunoreactivity; molecular testing (by TruSight RNA pan-cancer NGS) revealed FGFR3 (exon18)::TACC3 (exon8) fusion and no variant/alteration of IDH, BRAF, H3F3A or 1p/19q co-deletion.

Postoperative clinical assessment was unremarkable, and an MRI performed on postoperative day 2 showed expected postoperative changes with no evidence of residual tumor. He did not require adjuvant therapy post-resection and was followed with a serial MRI for surveillance.

Ten and a half months after his initial presentation, Mr. A developed a rapid onset of symptoms over 1–2 weeks, including left-sided headaches, right facial weakness, dysarthria, and dysphagia. He subsequently presented to the emergency department, and the CT head (Figure 2) revealed a new subtle hyperdense focus in the left frontal region adjacent to the body of the left lateral ventricle measuring 0.7 cm. This new lesion was distant from his previous right frontal lesion and craniotomy, and no evidence of PLNTY recurrence was appreciated. Subsequent MRI brain imaging performed several weeks later (Figure 2) showed an interval increase in the size of the lesion, described as a well-defined, oval FLAIR hyperintense lesion within the left centrum semiovale/corona radiata, measuring 1.9 × 1.4 × 1.7 cm. Multi-voxel spectroscopy demonstrated increased choline and decreased creatinine and NAA peaks, with a suspected mild increased lactate peak. On post-contrast images, diffuse patchy enhancement was noted with a focal area of more well-defined rim enhancement at the superomedial aspect of the lesion. Given these findings, the lesion was suspected to be a high-grade primary neoplasm. Mr. A’s case was discussed in a multidisciplinary setting, and the recommendation was made to proceed with a biopsy.

He underwent a left stereotactic biopsy of the left frontal lesion 416 days after his initial presentation with PLNTY and 97 days after developing symptoms related to the left frontal lesion. The pathology report confirmed a diagnosis of supratentorial ZFTA fusion-positive ependymoma, WHO grade 3. Specifically, the ZFTA (C11orf95-Ins125-RELA) fusion was detected by NanoString. The glioma DNA NGS revealed variants of TERT (c.-124C>T), TP53 (c.817C>A), and PTEN deletion, but no other variants including H3F3A.

**Figure 3:**
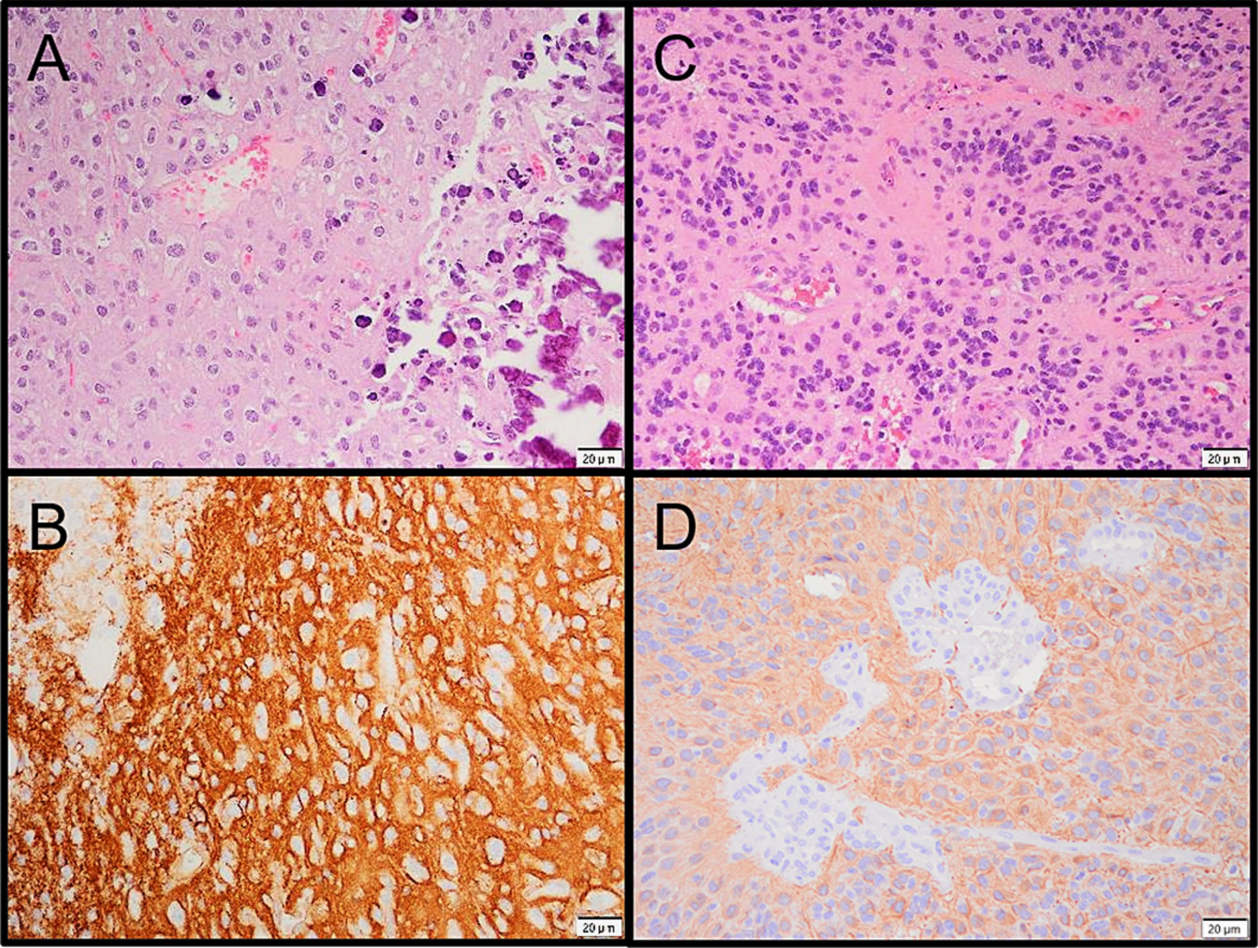
**(A)** Microphotograph of the PLNTY. **(B)** CD34 immunoreactivity in the PLNTY. **(C)** Microphotograph of the supratentorial ependymoma. **(D)** GFAP immunoreactivity in the ependymoma.

Based on this molecular finding, along with tumor’s histological and immunohistochemical characteristics, the lesion was further classified as a supratentorial ependymoma, ZFTA fusion-positive. Immunohistochemistry demonstrated largely positive GFAP staining (Figure 3) and diffusely positive p53 and p16. H3F3A mutations were not detected using molecular testing (Glioma DNA NGS).

**Figure 4:**
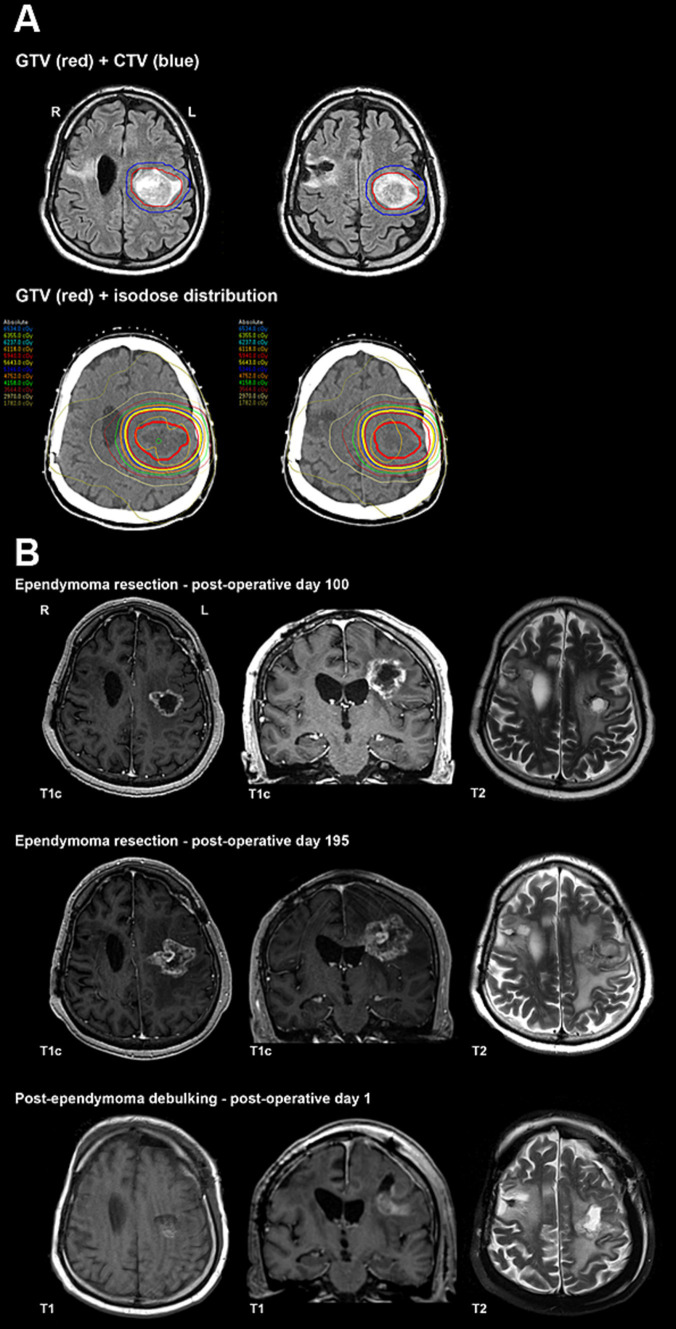
**(A)** Primary radiotherapy for ependymoma contours and isodose distribution. **(B)** Ependymoma post-operative imaging.

An MRI of the whole spine revealed no evidence of drop metastases. After further multidisciplinary discussions, the decision was made to proceed with primary radiotherapy (Figure 4A). Mr. A received 5,940 cGy in 33 fractions, completing treatment 184 days after he initially presented with symptoms related to supratentorial ependymoma.

After completing radiation therapy, Mr. A reported worsening headaches and increasing right-sided weakness. His post-radiation MRI raised concerns regarding recurrent or progressive neoplasm. Following further multidisciplinary discussion, resection of the left frontal ependymoma was performed 43 days after completing radiation. Pathological assessment of the surgical samples revealed predominantly necrotic tissue (approximately 70%) with small areas of viable tumor. The non-necrotic tissue contained a hypercellular tumor composed of markedly pleomorphic cells, including a few multinucleated cells and epithelioid cells with prominent nucleoli or nuclear inclusions/pseudo-inclusions. Molecular and immunohistochemistry testing results were similar to the pre-treatment biopsy, and a focally high Ki67 proliferation index was observed.

Repeat MRI with perfusion-weighted imaging was performed on postoperative day 100 (Figure 4B) and indicated a combination of residual/recurrent enhancing tumor and necrosis. Subsequent imaging on postoperative day 195 (Figure 4B) revealed an increased extent of enhancing tissue along with central enhancement, FLAIR abnormality, and mass effect, highly suspicious for tumor progression. No evidence of recurrence was appreciated in the resection area for the PLNTY lesion. After a multidisciplinary discussion, repeat resection was recommended.

Mr. A underwent a left frontal craniotomy for debulking and tumor resection 230 days after his previous surgery. Tissue examination revealed no classic histopathologic features of ependymoma but instead showed residual/recurrent glial neoplasm with extensive and dominant post-radiation and therapy-related effects. Postoperative MRI revealed expected postoperative changes and significant residual disease (Figure 4B).

Unfortunately, on postoperative day 4 following his third surgery, Mr. A developed a fever and experienced multiple focal and generalized tonic–clonic seizures. Despite aggressive treatment with antibiotics and antiseizure medications, his cognitive status did not return to baseline, and he continued to experience breakthrough seizures. In accordance with Mr. A’s previously expressed wishes, a palliative approach to care was adopted. He passed away in the hospital on postoperative day 17, 790 days after his initial presentation with symptoms associated with PLNTY.

## Discussion

In this case report, we discussed the unfortunate experience of a 41-year-old man who was sequentially diagnosed with two rare CNS tumors in less than a year: polymorphous low-grade neuroepithelial tumor of the young (PLNTY), followed by a grade 3 supratentorial ependymoma 10 months later. To the best of our knowledge, this is the first documented case of this dual diagnosis in the existing medical literature. His management involved a complex, multidisciplinary approach to diagnose and treat these conditions, but unfortunately, it proved fatal.

As previously discussed, PLNTY is a relatively new classification of low-grade neuroepithelial tumors, first described in 2017 (1). While a few case reports exist of PLNTY being diagnosed in adults (4, 5), none of these cases have demonstrated the occurrence of a second CNS malignancy. Typically, PLNTY presents with seizure (14), and this case is no exception. The precise reason for the tumor-type epileptogenic nature remains unclear but may be related to its frequent occurrence in the temporal lobes (14), its presentation during childhood when brain development is ongoing, or an inherent epileptogenic component of the tumor tissue itself. In this case, the tumor presented in the right frontal lobe of an adult, and its epileptogenic properties likely led to the initial seizure presentation. Notably, the patient remained seizure-free after the resection of the PLNTY until symptoms associated with the ependymoma emerged.

At present, no known genetic syndrome predisposes individuals to PLNTY, nor are there documented syndromes linking PLNTY and ependymoma. However, a review of the literature on current genetic biomarkers and immunohistochemical staining reveals two commonalities between PLNTY and ependymoma: mutations in the histone gene H3F3A and positive Glial Fibrillary Acidic Protein (GFAP) staining on immunohistochemistry (IHC).

Mutations in the H3F3A gene, particularly K27M, are common in childhood high-grade gliomas and have been identified in cases of PLNTY (15). Similarly, H3 K27M mutations have been reported in some ependymoma cases, although the data remain limited. A 2022 case series documented nine cases of pediatric posterior fossa ependymoma, all demonstrating the K27M mutation in the H3F3A gene (16). While this evidence is suggestive, it does not establish a genetic link between PLNTY and ependymoma. However, it highlights the potential for further investigations into H3 K27M mutations as a shared factor between these two diagnoses.

Additionally, exploring similarities in IHC staining, both PLNTY (1) and ependymoma (17, 18) are known to stain positive for GFAP. GFAP is an intermediate filament protein found in astrocytes within the CNS (19), and positive staining has long been established in CNS pathologies, including astrocytomas and gliomas (20). The recent finding of positive GFAP staining in both PLNTY and ependymoma suggests another avenue for exploring possible links between these two CNS pathologies.

In conclusion, we have presented a rare case of a 41-year-old man diagnosed sequentially with a polymorphous low-grade neuroepithelial tumor of the young, followed by a supratentorial grade 3 ependymoma. To the best of our knowledge, this unique dual pathology has not been previously described in the literature. The diagnosis and management of this case highlight the importance of multidisciplinary and multicenter collaboration in addressing complex and uncommon pathologies. At present, there is no known genetic diagnosis linking these two CNS pathologies. PLNTY cases are particularly rare, especially in adults, and supratentorial ependymomas account for only a minority of new ependymoma diagnoses in adults. Further research and collaborative efforts are needed to explore potential connections between these two distinct CNS pathologies.

## Data Availability

The original contributions presented in the study are included in the article/supplementary material, further inquiries can be directed to the corresponding author.
